# Intrathymic injection of hematopoietic progenitor cells establishes functional T cell development in a mouse model of severe combined immunodeficiency

**DOI:** 10.1186/s13045-017-0478-z

**Published:** 2017-05-16

**Authors:** Andrea Z. Tuckett, Raymond H. Thornton, Richard J. O’Reilly, Marcel R. M. van den Brink, Johannes L. Zakrzewski

**Affiliations:** 1 Department of Immunology, Memorial Sloan Kettering Cancer Center, 1275 York Avenue, New York, NY USA; 2 Department of Radiology, Memorial Sloan Kettering Cancer Center, 1275 York Avenue, New York, NY USA; 3 Department of Pediatrics, Memorial Sloan Kettering Cancer Center, 1275 York Avenue, New York, NY USA; 4 Department of Medicine, Memorial Sloan Kettering Cancer Center, 1275 York Avenue, New York, NY USA

**Keywords:** SCID, NSG mouse, Thymus, Cell therapy, Hematopoietic stem cell

## Abstract

**Background:**

Even though hematopoietic stem cell transplantation can be curative in patients with severe combined immunodeficiency, there is a need for additional strategies boosting T cell immunity in individuals suffering from genetic disorders of lymphoid development. Here we show that image-guided intrathymic injection of hematopoietic stem and progenitor cells in NOD-scid IL2rγ^null^ mice is feasible and facilitates the generation of functional T cells conferring protective immunity.

**Methods:**

Hematopoietic stem and progenitor cells were isolated from the bone marrow of healthy C57BL/6 mice (wild-type, Luciferase^+^, CD45.1^+^) and injected intravenously or intrathymically into both male and female, young or aged NOD-scid IL2rγ^null^ recipients. The in vivo fate of injected cells was analyzed by bioluminescence imaging and flow cytometry of thymus- and spleen-derived T cell populations. In addition to T cell reconstitution, we evaluated mice for evidence of immune dysregulation based on diabetes development and graft-versus-host disease. T cell immunity following intrathymic injection of hematopoietic stem and progenitor cells in NOD-scid IL2rγ^null^ mice was assessed in a B cell lymphoma model.

**Results:**

Despite the small size of the thymic remnant in NOD-scid IL2rγ^null^ mice, we were able to accomplish precise intrathymic delivery of hematopoietic stem and progenitor cells by ultrasound-guided injection. Thymic reconstitution following intrathymic injection of healthy allogeneic hematopoietic cells was most effective in young male recipients, indicating that even in the setting of severe immunodeficiency, sex and age are important variables for thymic function. Allogeneic T cells generated in intrathymically injected NOD-scid IL2rγ^null^ mice displayed anti-lymphoma activity in vivo, but we found no evidence for severe auto/alloreactivity in T cell-producing NOD-scid IL2rγ^null^ mice, suggesting that immune dysregulation is not a major concern.

**Conclusions:**

Our findings suggest that intrathymic injection of donor hematopoietic stem and progenitor cells is a safe and effective strategy to establish protective T cell immunity in a mouse model of severe combined immunodeficiency.

**Electronic supplementary material:**

The online version of this article (doi:10.1186/s13045-017-0478-z) contains supplementary material, which is available to authorized users.

## Background

T cell deficiency presents a major clinical challenge in severe combined immunodeficiency (SCID) patients. SCID comprises a variety of genetic disorders of lymphoid development. These genetic defects primarily affect hematopoietic stem cells in the bone marrow by impairing their ability to differentiate into lymphocytes. The thymus, the organ where T lymphocytes develop from bone marrow-derived hematopoietic progenitor cells, is atrophied from lack of being seeded with healthy hematopoietic progenitor cells, but thymic stromal and epithelial cells are genetically normal in SCID patients. However, it is well known that crosstalk between developing T cells and thymic epithelial cells is of critical importance for normal thymic function [1] and lack thereof over long periods of time will result in significant compromise of the thymic microarchitecture and of thymic epithelial cell survival and function [2]. Introduction of normal hematopoietic progenitor cells into the thymic microenvironment of SCID patients can therefore establish variable levels of thymopoiesis, but the likelihood of success inversely correlates with the patient’s age [3]. While allogeneic hematopoietic stem cell transplantation is the standard of care for the treatment of SCID, Taylor and coworkers explored a possible role for intrathymic injection of lineage marker-negative (Lin^−^) bone marrow cells in ZAP-70-deficient hosts [4–6], a condition associated with lack of functional mature T cells [7]. The most common genetic defects in SCID include deficiency of IL2rγ, RAG1/2, or adenosine deaminase, among others [8]. We sought to investigate if intrathymic injection could be of value in a mouse model associated with one of the most severe forms of immunodeficiency, the NOD-scid IL2rγ^null^ (NSG) mouse [9]. Due to their profound immunodeficiency, NSG mice are frequently used as hosts in tumor xenograft models, and humanized NSG mice are a popular platform for immunology, immuno-oncology, and infectious disease research. NSG mice are characterized by complete absence of mature T, B, and NK cells. Of note, endogenous T cell development in NSG mice is not completely abrogated since combining SCID with γ_c_ deficiency is still permissive for generation of very small numbers of the earliest T cell progenitor stages [10]. The thymic remnant in NSG mice measures only about 1 mm^3^ [11], requiring image guidance to ensure accurate injection of the target. We therefore utilized our previously developed minimally invasive method for ultrasound-guided intrathymic injection [11, 12] of hematopoietic stem/progenitor cells (HSPCs) to establish T cell development in NSG mice. We characterized thymic and splenic T cell subsets in male and female as well as young and aged mice, and we assessed in vivo T cell function in NSG mice following intrathymic injection of HSPCs.

## Methods

### Mice

Non-obese diabetic (NOD) mice [13] were purchased from the Jackson Laboratory; NSG mice were obtained from the Mouse Genetics Core Facility at Memorial Sloan Kettering Cancer Center (MSKCC). C57BL/6 mice were purchased from the Jackson Laboratory. C57BL/6.luc^+^ (Luciferase^+^) transgenic mice obtained from Robert Negrin (Stanford University) [14] were generated and maintained at MSKCC. Unless otherwise indicated, 8- to 12-week-old male mice were used as both donors and hosts. All animal work was performed in compliance with MSKCC Institutional Animal Care and Use Committee (IACUC) protocols.

### Cells

HSPCs for intrathymic or intravenous injection were isolated from the bone marrow of C57BL/6 mice by magnetic bead-mediated enrichment of Lin^−^ cells followed by flow cytometric sorting of Lin^−^Sca-1^+^c-kit^+^ (LSK) cells. Luciferase-expressing A20-TGL mouse lymphoma cells on a BALB/c background (H-2^d^) [15] were cultured in RPMI supplemented with 10% FCS and 1% penicillin/streptomycin.

### Intrathymic injection

Aseptic ultrasound-guided intrathymic injections were performed as described previously [11], using 30-gauge insulin syringes and the Vevo 2100 imaging system (VisualSonics) with a MS-550S 40-mHz linear transducer. A minimum of 2,000 and up to 10,000 cells resuspended in 10 μl of phosphate-buffered saline (PBS) were injected.

### In vivo bioluminescent imaging

Intravital monitoring of intrathymically injected LSK cells or intravenously injected A20-TGL lymphoma cells was performed by in vivo bioluminescent imaging (BLI). Mice received d-luciferin (3 mg/mouse; GoldBio) intraperitoneally, were anesthetized by isoflurane and were imaged using the IVIS Spectrum imaging system (PerkinElmer).

### Histology

For histological analyses including H&E staining, immunohistochemistry and immunofluorescence, samples were fixed in 4% paraformaldehyde, embedded in paraffin, sectioned, stained, and then evaluated by microscopic imaging.

### Blood glucose testing

Glucose monitoring of tail vein blood from NSG mice was performed with a Precision Xtra system (Abbott Diabetes Care Inc.). Blood was assayed from day −1 to day 149 post injections.

### Flow cytometry

Cells were incubated with antibodies for 15 min at 4 °C and washed twice. The stained cells were resuspended in buffer and analyzed on an LSR II flow cytometer (Becton Dickinson) with FACSDiva software (Becton Dickinson). Data were analyzed with FlowJo software (Tree Star).

### Reagents and antibodies

All of the following monoclonal antibodies against murine antigens were obtained from BD Biosciences: CD3, CD4, CD8a, CD19, CD62L, CD44, NK1.1, CD11c, CD11b, Gr-1, c-kit, Sca-1, mouse Vβ TCR screening panel. A mouse FoxP3-specific antibody was purchased from eBioscience, and a mouse Helios-specific antibody was purchased from BioLegend. A fixation/permeabilization kit was also obtained from eBioscience. Diamidino-phenylindole (DAPI) (Molecular Probes) was used for dead cell discrimination.

### Statistics

All results in this manuscript were based on two-sided test statistics. A *P* value <0.05 was considered statistically significant. The Mann-Whitney *U*-statistic was used to compare data between two groups. Statistical analysis was performed using Prism software (GraphPad). All experiments were repeated at least once.

## Results

### Intrathymic hematopoietic stem cell transfer establishes T cell generation in NSG mice

**Fig. 1:**
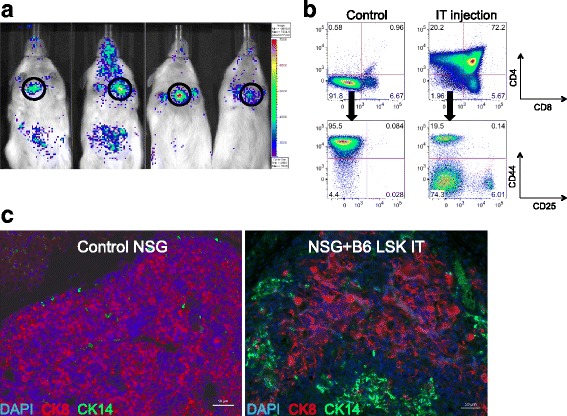
Intrathymic injection of hematopoietic stem/progenitor cells induces a functional thymic microenvironment in NSG mice. **a** NSG recipients were intrathymically injected with 10,000 luciferase-expressing C57BL/6 LSK cells. The whole-body distribution of LSK-derived cells on day 15 after injection was monitored by in vivo BLI. Circles highlight the concentration of injected cells within the thymic area. Pseudocolor images superimposed on conventional photographs are shown. Four representative animals of ten are presented. **b** NSG recipients were intrathymically injected with 2000 C57BL/6 LSK cells. Thymuses were harvested 1 month after injection and analyzed for thymocyte subsets (DP, CD4 SP, CD8 SP, DN) (*upper panel*) and DN subsets (DN 1, 2, 3 and 4 subsets defined by expression of CD25 and CD44) (*lower panel*) by multicolor flow cytometry. Plots for one of three mice are presented. The thymus of an untreated NSG mouse was used as the control. **c** NSG mice were intrathymically injected with 10,000 luciferase-expressing C57BL/6 LSK cells. The thymus of an untreated NSG mouse was used as the control. Thymuses were harvested at day 60 and analyzed for cytokeratin (CK) 8 (*red*), CK 14 (*green*), and DAPI (*blue*) by immunofluorescence. Images were acquired with an Axio Imager (Zeiss) and processed with Zeiss ZEN imaging software. *Scale bar* 50 μm. One representative image is shown (*n* = 4). DP: CD4 and CD8 double-positive cells; DN: CD4 and CD8 double-negative cells; CD4 SP: CD4 single-positive cells; CD8 SP: CD8 single-positive cells

**Fig. 2:**
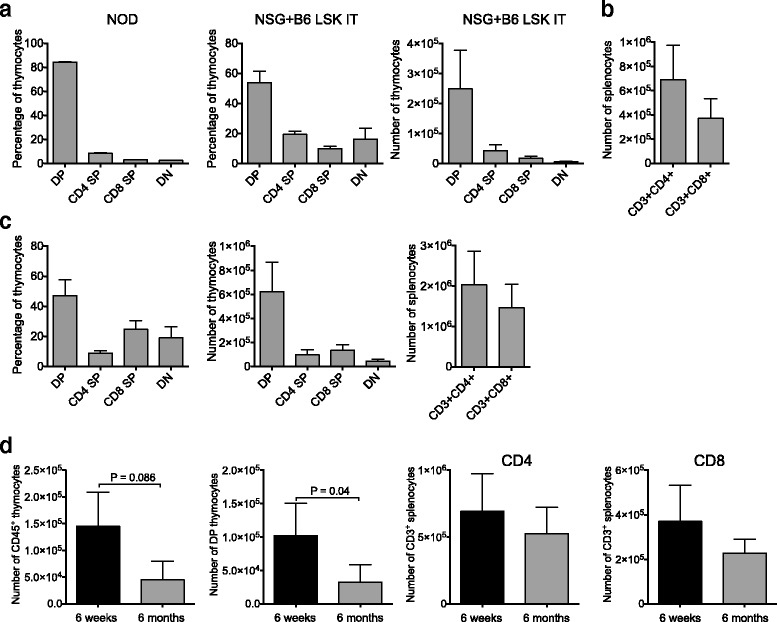
Intrathymic injection of hematopoietic stem/progenitor cells establishes thymopoiesis in young and aged NSG mice. **a** NSG mice were intrathymically injected with 10,000 C57BL/6 LSK cells. Thymuses were harvested on day 56 and analyzed by flow cytometry. *Left panel* shows thymocyte subsets for untreated NOD mice included as a reference group. *Center* and *right panels* show frequency and absolute numbers for thymocyte subsets of injected NSG mice. Data combined from two independent experiments are shown. Mean and SEM are presented (*n* = 8–12). **b** NSG mice were treated as in (**a**). Spleens were harvested on day 56 and T cells were analyzed by flow cytometry. Combined data from two independent experiments are shown. Mean and SEM are presented (*n* = 8–10). **c** Mice were treated as in (**a**). Thymuses and spleens were harvested 5 months after injection and analyzed by flow cytometry. *Left panel* shows frequency of thymocyte subsets; *center panel* shows absolute numbers of thymocyte subsets, and *right panel* shows absolute numbers of splenic T cells. Data combined from two independent experiments are shown. Mean and SEM are presented (*n* = 8–12). **d** Mice were treated as in (**a**) for aged mice (6 months old) and young mice (6 weeks old). Thymuses and spleens were harvested at day 56. *Panels 1 and 2*: absolute numbers of thymocytes and DP thymocytes in young versus aged mice. *Panels 3 and 4*: absolute numbers of CD4^+^ and CD8^+^ splenic T cells in young versus aged mice. Data combined from two independent experiments are shown. Mean and SEM are presented (*n* = 8–12)

**Fig. 3:**
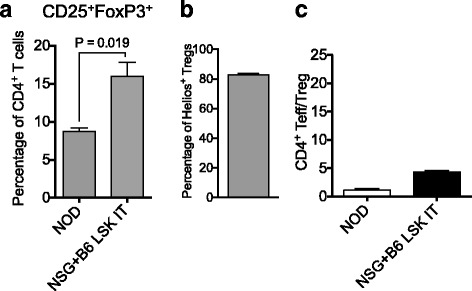
The frequency of regulatory T cells is increased in CD4^+^ T cells developing in NSG mice following intrathymic injection of hematopoietic stem/progenitor cells. NSG mice were intrathymically injected with 10,000 C57BL/6 LSK cells. Spleens were harvested on day 56 and CD4^+^ T cell subsets were analyzed by flow cytometry. **a** Splenic CD25^+^FoxP3^+^ regulatory T cells (frequencies of CD4^+^ T cells) in intrathymically injected NSG mice and NOD control, untreated mice. Combined data from two independent experiments are shown. Mean and SEM are presented (*n* = 5–10). **b** The frequencies of Helios^+^ natural regulatory T cells that were generated by intrathymically injected NSG mice. Mean and SEM are presented (*n* = 9). **c** The ratio of CD4^+^ effector (CD62L^−^CD44^+^)/regulatory T cells in NOD control mice and intrathymically injected NSG mice. Mean and SEM are presented (*n* = 5–13)

**Fig. 4:**
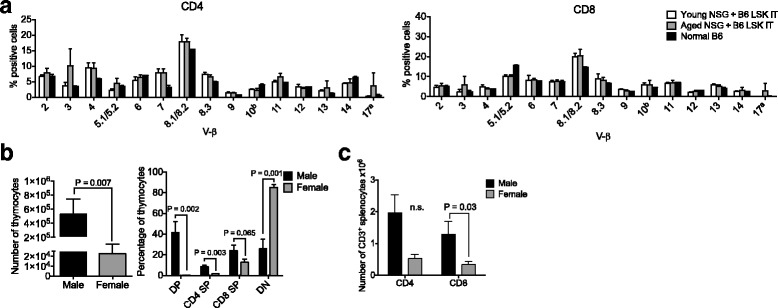
T cells derived from intrathymically injected NSG hosts are phenotypically and functionally similar to normal T cells. **a** Young versus aged NSG recipients were intrathymically injected with 10,000 C57BL/6 LSK cells. Spleens were harvested on day 56 and TCR Vβ families were analyzed by multicolor flow cytometry. Mean and SEM are presented (*n* = 5). Two normal C57BL/6 mice were included as control. **b** Female and male NSG hosts were intrathymically injected with 10,000 C57BL/6 LSK cells. Thymuses were harvested 5 months after injection. Thymocyte counts, frequency of thymocyte subsets, and absolute values of splenic T cell subsets were analyzed by multicolor flow cytometry. Mean and SEM are presented (*n* = 11–13). **c** NSG mice were treated as described in **b**. Spleens were harvested 5 months after injection and T cells were analyzed by flow cytometry. Mean and SEM are presented (*n* = 11–13)

We previously reported that intrathymic injection of NSG recipients was feasible when performed with ultrasound guidance [11]. We applied this method to the setting of adoptive cell therapy by isolating LSK cells, a population comprising hematopoietic stem as well as committed progenitor cells, from the bone marrow of C57BL/6 mice and injecting them under direct visualization (ultrasound guidance) into the thymus of male NSG recipients. Engraftment and expansion of injected cells was monitored by in vivo BLI (Fig. 1a and data not shown). Thymic engraftment and a donor cell phenotype consistent with thymic T cell development (Fig. 1b) as well as the presence of thymic cortical (CK8^+^) and medullary (CK14^+^) epithelial cells (Fig. 1c) were confirmed in tissues harvested several weeks after injection. Of note, the thymic remnant of non-treated control NSG mice was characterized by a complete lack of a thymic medullary region (Fig. 1c). Figure 2a–c shows the kinetics of T cell reconstitution in intrathymically injected NSG mice. We did not include a control group depicting non-injected NSG mice due to the complete absence of T cells in these mice. The frequency of thymocyte subsets in NSG mice 2 months after injection resembled that of euthymic mice such as NOD mice that were included as a reference group (Fig. 2a, panels 1 and 2). Mean thymic cellularity 8 weeks after injection reached only about 300,000 cells despite injection of high cell doses (10,000 LSK cells) (Fig. 2a, panel 3). Splenic CD4 and CD8 T cell numbers amounted to 400,000 and 600,000 cells on average 2 months after injection (Fig. 2b). Thymic cellularity as well as the splenic T cell numbers further increased between months 2 and 5 after injection (Fig. 2c). In order to assess whether age-related thymic involution played a meaningful role in NSG mice, we compared 6-week-old with 6-month-old NSG recipients of intrathymically injected C57BL/6 LSK cells. We found that 2 months after injection, thymic cellularity, in particular the number of double-positive thymocytes, was decreased in aged NSG mice (Fig. 2d, panels 1 and 2), but there was no significant difference in the number of splenic CD4 and CD8 T cells (Fig. 2d, panels 3 and 4). Of note, the frequency of regulatory T cells (Tregs) among CD4^+^ T cells was significantly increased in injected NSG mice compared to NOD controls (Fig. 3a), which is most likely the result of increased thymic generation of Helios^+^ natural Tregs (Fig. 3b). The ratio of effector T cells to Tregs of CD4^+^ T cells typically increases with the degree of immune activation. We found that this ratio was only slightly increased in T cells derived from NSG mice that were intrathymically injected with C57BL/6 LSK cells compared to NOD control T cells (Fig. 3c), and it was about 20 times lower than the range that has been reported for the setting of severe immune activation such as allogeneic transplantation [16]. Progeny of intrathymically injected C57BL/6 LSK cells were characterized by a diverse T cell receptor repertoire similar to that of normal C57BL/6 T cells in both young and aged NSG recipients (Fig. 4a).

### Thymopoiesis is gender dependent but generated T cells do not display signs of severe immune dysregulation

**Fig. 5:**
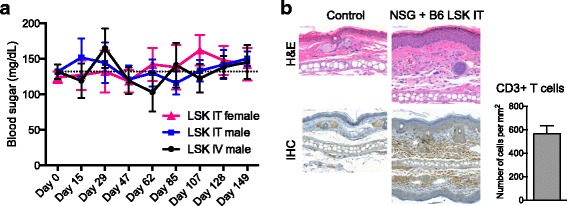
There is no evidence for major immune dysregulation in NSG mice that were intrathymically injected with hematopoietic stem/progenitor cells. **a** Female or male NSG recipients were injected intrathymically (*IT*) or intravenously (*IV*) with 10,000 C57BL/6 LSK cells. Non-fasting blood glucose readings were acquired from day −1 to day 149 using a glucometer. Mean and SEM are presented (*n* = 8–10). **b** Male NSG mice were intrathymically injected with 10,000 C57BL/6 LSK cells. Ears were clipped at 1 month after injection and analyzed histologically by H&E staining (*upper panels*) and immunohistochemistry (*IHC*) for CD3 (*lower panels*). Tissue sections were scanned with a Mirax Micro digital slide scanner (Zeiss) and viewed with Panoramic Viewer software (3DHISTECH Ltd.). *Scale bar left panels* and *top right panel* 50 μm; *scale bar bottom center panel* 100 μm. One representative image is shown (*n* = 3). An age- and sex-matched untreated NSG mouse was included as control. *Bottom right panel*: mean and SEM of T cell counts based on CD3 IHC analysis of treated NSG mice are presented. Cell numbers were determined by quantification of the CD3 staining with ImageJ software; measurements were corrected for background signal (based on untreated control NSG mice) (*n* = 3)

Next, we compared male and female NSG recipients of intrathymically injected LSK cells to be able to assess possible sex-specific disparities regarding thymic reconstitution. This question is of particular concern in the context of mice on a NOD background since the NOD mouse represents a well-established autoimmunity model [17], and immune dysregulation is more pronounced in female compared with male NOD mice as evidenced by earlier onset and higher incidence of diabetes [15, 18]. We found that thymic reconstitution and splenic T cell numbers were significantly increased in male compared with female recipients 5 months after injection (even though the increase in splenic CD4^+^ T cell numbers did not reach statistical significance) (Fig. 4b, c). In order to screen animals for diabetes, we compared non-fasting blood sugar levels in male and female NSG recipients of intrathymically injected LSK cells; we included intravenously injected male recipients as a reference group to control for possible differences in thymic tolerance induction in intravenously versus intrathymically injected recipients (Fig. 5a). We found that blood glucose levels fluctuated with episodes of mild hyperglycemia in all groups but no animal developed overt diabetes (as defined by two consecutive non-fasting blood glucose levels of at least 250 mg/dl [15, 17]) throughout a 5-month follow-up period. We next assessed male and female recipients of intrathymically injected allogeneic LSK cells for evidence of graft-versus-host disease (GVHD). We did not observe any weight loss or other clinical signs of GVHD with the exception of mild skin inflammation in about one third of the mice of both sexes. Low-grade skin GVHD in this minority subset of recipients was confirmed by histology (Fig. 5b and data not shown).

### T cells generated by intrathymically injected NSG mice exert potent in vivo activity

**Fig. 6:**
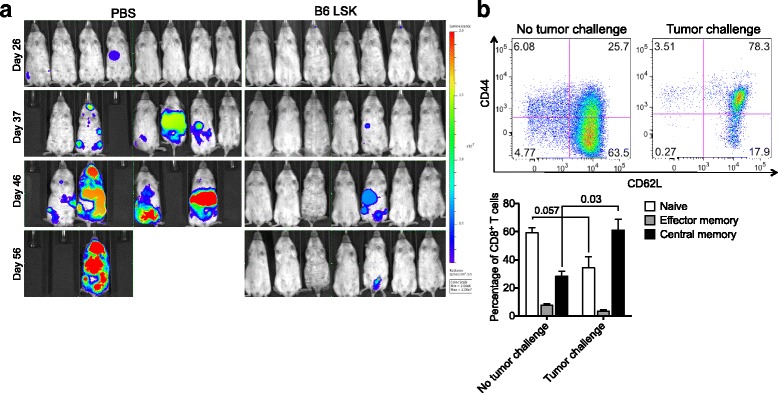
T cells developing in intrathymically injected NSG hosts display anti-lymphoma activity. **a** NSG mice were intrathymically injected with 10,000 C57BL/6 LSK cells or PBS. Five weeks after intrathymic injection, A20-TGL mouse lymphoma cells were injected intravenously into all mice. The whole body distribution of A20-TGL luciferase-expressing cells was monitored by in vivo BLI. Pseudocolor images superimposed on conventional photographs are shown (*n* = 7–8). **b** Mice in the tumor challenge group were treated as in(**a**). Spleens were harvested 2 months post tumor challenge. Mice in the no tumor challenge group were treated as follows: NSG mice were intrathymically injected with 10,000 C57BL/6 LSK cells. No A20-TGL cells were administered. Spleens were harvested on day 56 after LSK cell injections. In both groups, splenic CD8^+^ T cells were analyzed for expression of CD62L and CD44 by multicolor flow cytometry. Naïve: CD62L^+^CD44^−^; central memory: CD62L^+^CD44^+^; effector memory: CD62L^−^CD44^+^. One representative dot plot is shown. Mean and SEM are presented (*n* = 6)

Finally, we analyzed protective immunity mediated by T cells generated by intrathymic LSK cell injection in NSG mice. To this end, we challenged NSG mice with tumor cells sensitive to T cell killing (A20 lymphoma) 5 weeks after intrathymic injection of LSK cells versus PBS. LSK cell intrathymic injection prevented establishment of this aggressive lymphoma in all recipients (Fig. 6a and Additional file 1), which is remarkable considering the limited thymopoiesis that is possible in NSG mice following intrathymic injection of HSPCs. Moreover, comparing the naïve and memory phenotypes of CD8 T cells in intrathymically injected NSG mice with and without tumor challenge was consistent with a T cell effector response to the lymphoma challenge (Fig. 6b).

## Discussion

Several preclinical studies have explored possible therapeutic benefits of cell therapy based on intrathymic delivery of hematopoietic cells to either irradiated wild-type recipients [12] or ZAP-70-deficient hosts [4–6]. In mice with the most severe defects of lymphoid development such as NSG mice, the thymic remnant is so small that image guidance is required for accurate intrathymic injection. We therefore utilized a cell delivery method based on ultrasound-guided free-hand injection to show that T cell generation can be induced in NSG mice by intrathymic injection of healthy allogeneic HSPCs, underscoring the potential usefulness of intrathymic cell therapy in the setting of SCID.

We show that the thymic remnant in NSG mice is characterized by the presence of cortical thymic epithelial cells in the absence of a medulla. Of note, the initial development and formation of a thymic epithelial microenvironment does not depend on the presence of thymocytes [19]. However, thymocyte-derived signals are required for maintenance of established mature thymic epithelial cells [20]. Our findings suggest that the thymic cortex but not the medulla can be maintained in the prolonged absence of thymocytes. Moreover, based on our results and consistent with previous studies in immunodeficient mice [20, 21], we hypothesize that postnatal induction of T cell development in the residual thymic cortex of NSG mice can restore formation of a thymic medullary microenvironment. Not surprisingly, intrathymic injection of normal HSPCs into adult male NSG mice did not restore thymic cellularity to the level of normal mice, which is in the order of tens of millions of thymocytes, compared to less than one million in intrathymically injected NSG mice. However, despite the limited regenerative capacity of the thymus, the number of splenic T cells was still able to reach biologically meaningful levels in NSG mice that were intrathymically injected with normal HSPCs. We observed a clear age effect on the thymic responsiveness to intrathymic injection of normal HSPCs, and while we did not perform intrathymic injections in newborn mice, it is very likely that the best outcomes would be achievable during the first weeks of life when the chances are highest that damages to the thymic microenvironment can be reversed. Comparison of male and female mice revealed a striking difference in the regenerative capacity of the thymus in favor of male recipients, suggestive of a differential effect of male versus female sex steroids on thymic function in NSG mice. This observation underscores the importance of the consideration of sex as a biological variable, and consideration of this phenomenon should be of particular importance when utilizing humanized NSG mice for immune reconstitution studies. Our data with regard to T cell differentiation (Treg/T effector cell balance) and GVHD indicate that progeny of intrathymically injected allogeneic HSPCs is appropriately tolerized during thymic T cell development and therefore does not trigger severe GVHD. Moreover, we did not observe diabetes development—the hallmark of immune dysregulation in NOD mice—in NSG mice injected with healthy HSPCs. The previously described increase in Tregs (Fig. 3a) may be a factor contributing to the absence of diabetes induction in T cell-competent NSG mice [22, 23]. In addition, the absence of diabetes in NSG mice injected with normal HSPCs likely reflects the lack of previously reported T cell-intrinsic immune dysregulation that is mediated by T cells of NOD background (but not by wild-type T cells) [24]. Finally, using a B cell lymphoma model as readout for in vivo T cell function, we were able to demonstrate that C57BL/6 T cells developing in intrathymically injected NSG mice were able to engage and kill B cell lymphoma cells in vivo. This indicates that wild-type T cells generated by the thymus of NSG mice provide beneficial protective immunity and are not prone to significant alloreactivity or autoreactivity.

## Conclusions

Taken together, our findings confirm that intrathymic injection of HSPCs should be considered safe and relatively effective in preclinical SCID models, especially when performed in male recipients. Immune dysregulation, a theoretical concern owed to the NOD background of NSG mice, is mild at best and does not appear to have major health implications in NSG mice transplanted with normal hematopoietic stem cells. Hematopoietic stem cell transplantation including approaches utilizing gene therapy [25] will continue to be the mainstay of therapy for SCID patients; however, based on the preclinical evidence provided by us and others [4–6], it is reasonable to suggest that in some patients with refractory T cell deficiency, there may be a role for intrathymic injection-based cell therapy as an additional therapeutic avenue.

## Additional file

Additional file 1: Intrathymically injected NSG mice survive A20 lymphoma challenge. (PDF 68 kb)

## Abbreviations

BLI: In vivo bioluminescence imaging
GVHD: Graft-versus-host disease
HSPC: Hematopoietic stem/progenitor cell
LSK: Lin^−^Sca-1^+^c-kit^+^
NSG: NOD-scid IL2rγ^null^
SCID: Severe combined immunodeficiency
Tregs: Regulatory T cells
