# Supplementary material for: Intrathymic injection of hematopoietic progenitor cells establishes functional T cell development in a mouse model of severe combined immunodeficiency
Source: J Hematol Oncol. 2017 May 16;10:109. doi: 10.1186/s13045-017-0478-z (PMC5432974; doi:10.1186/s13045-017-0478-z)
Supplement: Additional file 1: — Intrathymically injected NSG mice survive A20 lymphoma challenge. (PDF 68 kb) [file 13045_2017_478_MOESM1_ESM.pdf]

Intrathymically injected NSG mice survive A20 lymphoma challenge.

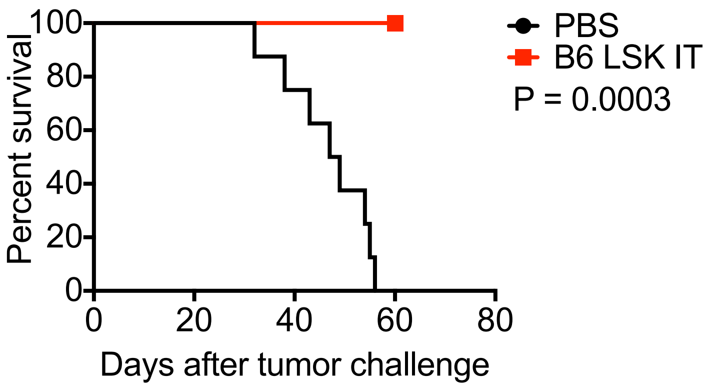

NSG mice were intrathymically injected with 10,000 C57BL/6 LSK cells or PBS. Five weeks after intrathymic injection A20-TGL mouse lymphoma cells were injected intravenously into all mice. Survival curve is presented; P value was determined by Log-rank test (n=7-8).
